# Optical Genome Mapping for Chromosomal Aberrations Detection—False-Negative Results and Contributing Factors

**DOI:** 10.3390/diagnostics14020165

**Published:** 2024-01-11

**Authors:** Yiyun Xu, Qinxin Zhang, Yan Wang, Ran Zhou, Xiuqing Ji, Lulu Meng, Chunyu Luo, An Liu, Jiao Jiao, Hao Chen, Huasha Zeng, Ping Hu, Zhengfeng Xu

**Affiliations:** Department of Prenatal Diagnosis, Women’s Hospital of Nanjing Medical University, Nanjing Women and Children’s Health Care Hospital, Nanjing 210004, China; xuyy321@163.com (Y.X.); njfybjyzhangqinxin@163.com (Q.Z.); wangyan@njmu.edu.cn (Y.W.); zhouran1021@163.com (R.Z.); yousheng66@sina.com (X.J.); menglu_12@126.com (L.M.); lcy0929@126.com (C.L.); fyann1213@163.com (A.L.); jiaojiao@stu.njmu.edu.cn (J.J.); 2022110719@stu.njmu.edu.cn (H.C.); huasha_zeng@163.com (H.Z.)

**Keywords:** optical genome mapping, structural variation, copy number variation, false-negative, segmental duplication

## Abstract

Optical genome mapping (OGM) has been known as an all-in-one technology for chromosomal aberration detection. However, there are also aberrations beyond the detection range of OGM. This study aimed to report the aberrations missed by OGM and analyze the contributing factors. OGM was performed by taking both GRCh37 and GRCh38 as reference genomes. The OGM results were analyzed in blinded fashion and compared to standard assays. Quality control (QC) metrics, sample types, reference genome, effective coverage and classes and locations of aberrations were then analyzed. In total, 154 clinically reported variations from 123 samples were investigated. OGM failed to detect 10 (6.5%, 10/154) aberrations with GRCh37 assembly, including five copy number variations (CNVs), two submicroscopic balanced translocations, two pericentric inversion and one isochromosome (mosaicism). All the samples passed pre-analytical and analytical QC. With GRCh38 assembly, the false-negative rate of OGM fell to 4.5% (7/154). The breakpoints of the CNVs, balanced translocations and inversions undetected by OGM were located in segmental duplication (SD) regions or regions with no DLE-1 label. In conclusion, besides variations with centromeric breakpoints, structural variations (SVs) with breakpoints located in large repetitive sequences may also be missed by OGM. GRCh38 is recommended as the reference genome when OGM is performed. Our results highlight the necessity of fully understanding the detection range and limitation of OGM in clinical practice.

## 1. Introduction

Genome structural variations, such as CNVs, translocations and inversions, are closely related to gene expression and contribute substantially to disease or disease susceptibility [1]. It is challenging to comprehensively characterize SVs, because SVs are highly enriched within or near repetitive DNA [2]. Nowadays, SVs are mainly identified by karyotyping, CMA and FISH. However, these technologies sometimes need to be performed sequentially or simultaneously for comprehensive assessment. Furthermore, some small (<50 kbp) SVs are beyond the detection scope of these technologies. Therefore, OGM, an all-in-one genetic test for SV detection, has received widespread attention.

OGM is an emerging and promising cytogenomic technology that has the potential to detect major classes of SVs [3]. Combining microfluidics, automated image analysis and high-resolution microscopy, OGM allows us to detect insertions and deletions above 500 bp in length and other SVs above 30 kbp in length [4]. There are two distinct algorithms. The CNV algorithm is based on analysis of normalized molecular coverage to detect aneuploidies and CNV > 500 kbp. The SV algorithm compares the sample labelling maps with the reference maps to identify SVs [5]. The efficacy and application value of OGM is still under assessment.

Recently, some research has investigated the ability of OGM to detect chromosomal aberrations [5,6]. OGM demonstrated a near-perfect performance that suggested OGM has the potential to be an alternative to conventional technologies for detecting clinically relevant aberrations comprehensively. Meanwhile, the limitations of OGM emerged. There were also some false-negative OGM results [7,8]. Therefore, it is necessary to fully understand the detection range and limitation of OGM before clinical application. It is also important to analyze the factors contributing to the false-negative results. However, due to the limited amount of research on OGM, the underlying contributing factors of false-negative results have not been clarified.

Herein, we performed OGM in 123 individuals with 154 clinically reported variations and highlighted the aberrations missed by OGM. We then analyzed the quality control metrics, sample types, reference genome, effective coverage and classes and locations of aberrations, and we tried to figure out the possible reasons leading to the false-negative results of OGM.

## 2. Materials and Methods

### 2.1. Subjcts

**Figure 1:**
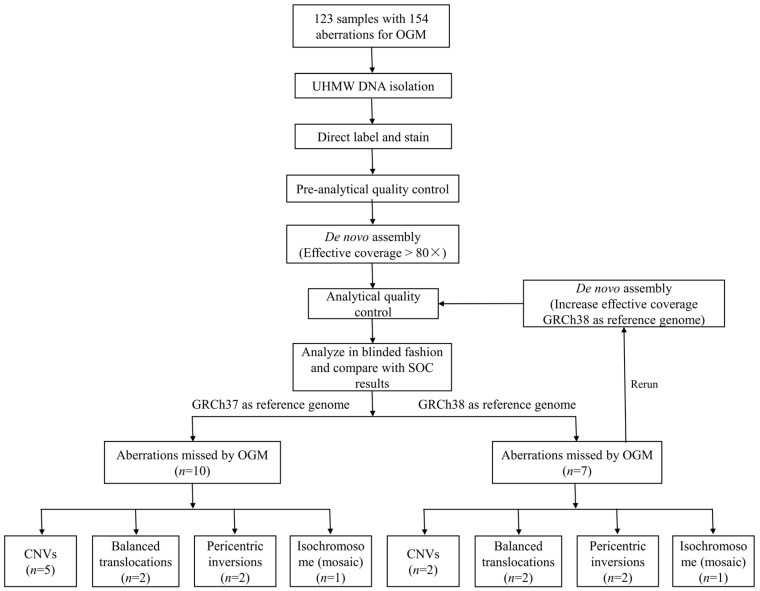
Flowchart of this study. OGM: optical genome mapping; UHMW: ultra-high molecular weight; SOC: standard-of-care; CNV: copy number variation.

The flowchart of our study is shown in Figure 1. A total of 123 individuals (109 prenatal cases and 14 postpartum cases) who underwent both OGM and SOC methods between 2022 to 2023, and were confirmed to harbor clinically reported aberrations, were included in this study. Informed consent was obtained from all the volunteers or their families at pre-test counseling. Our study was approved by the Medical Ethics Committee of Nanjing Maternity and Child Health Care Hospital (2021KY-118). 

### 2.2. Procedures

OGM was performed in all cases, taking both GRCh37 and GRCh38 as reference genomes. Results of SOC methods such as CMA, karyotyping, FISH (fluorescence in situ hybridization), MLPA (multiplex ligation-dependent probe amplification) and TP-PCR (Triple repeat- primed PCR) were collected to compare with that of OGM to figure out the aberrations missed by OGM. Clinically reported aberrations in our study included pathogenic or likely pathogenic (P/LP) aberrations (including aneuploidies, P/LP SVs, ROH (regions of homozygosity) located on chromosome 6, 7, 11, 14, 15 and 20 and FMR1 gene full mutation), variants of uncertain significance (VOUS) (duplications > 1 Mb, deletions > 500 kbp) and clinically significant balanced chromosome rearrangements.

#### 2.2.1. Optical Genome Mapping

For prenatal cases, approximately 5 mL amniotic fluid was collected and cultured in one T25 culture flask. The cultured cells were passaged on the 9 day and harvested at 80–90% confluence. About 1–2 million amniotic fluid cells of each prenatal sample were cryopreserved at −80 °C using 10% DMSO. For postpartum cases, 2 mL peripheral blood samples were collected in ethylenediamine tetraacetic acid (EDTA) vacutainer blood collection tube and froze at −80 °C or directly stored at 4 °C for ultra-high molecular weight (UHMW) DNA isolation.

Following the standard operation procedure, the main steps of OGM experiment include UHMW DNA isolation, direct label and stain and chip loading, as previously reported [9]. Firstly, UHMW DNA was isolated from amniotic fluid cells or peripheral blood according to the instruction of Bionano PrepTM Blood and cell culture DNA Isolation Kit (Bionano Genomics, San Diego, CA, USA). Qubit dsDNA BR assay kit (Thermo Fisher Scientific, Waltham, MA, USA) was used to conduct DNA quantification. A suitable DNA concentration range (36–150 ng/μL) was vital for subsequent experiment. Then, a total of 750 ng genomic DNA was labeled by enzyme DLE-1 following the instruction of Bionano Prep DLS Kit (Bionano Genomics, San Diego, CA, USA). The labeled UHMW DNA was quantified with Qubit dsDNA HS (high sensitivity) Assay Kit and Qubit Fluorometer. The labeled DNA concentration should be in the range of 4–12 ng/μl. After passing the pre-analytical quality control, the fluorescently labeled UHMW DNA was loaded onto the Saphyr Chip (Bionano Genomics, USA) and was linearized and imaged on the Saphyr instrument (Bionano Genomics, USA). Saphyr system performed de novo genome assembly intended to obtain the recommended effective genome coverage (80×). When aberrations were missed by OGM taking GRCh38 as reference genome, de novo genome assembly was performed again, aiming at increasing effective genome coverage.

OGM data were analyzed using Bionano Access (Ver. 1.7). Analytical quality control targets were set to achieve >80× effective genome coverage, >70% mapping rate, 14 to 17 label density (labels per 100 kbp) and >230 kbp N50 (of molecules > 150 kbp). Two algorithms (CNV algorithm and SV algorithm) were mainly applied to detecting different classes of variations.

The filtering thresholds of CNV algorithm were confidence > 0.95; size > 500 kbp; deletions with fractional copy number (FCN) < 1.2 and duplications with FCN > 2.8, as well as turning off the mask filter. The filtering thresholds of SV algorithm were as follows: turning off the mask filter; appearing in < 1% of the OGM internal control database; insertions and deletions (confidence > 0); inversions (confidence > 0.7); duplications (confidence > −1); intrachromosomal translocations (confidence > 0.05); interchromosomal translocations (confidence > 0.05). Aneuploidies and CNVs called by CNV algorithm and SVs called by SV algorithm were reviewed and classified according to American College of Medical Genetics and Genomics (ACMG) guidelines [10]. Additionally, ROH was called by ROH algorithm combined with manual inspection of VAF (variants allele fraction) pattern. Genomic variants consistent with FXS (fragile X syndrome) and FSHD1 (facioscapulohumeral muscular dystrophy type 1) were accessed using Bionano EnFocus pipelines. For FXS analysis, the size of the CGG repeats was inferred based on the measured distance between two neighboring labels on the assembled map that contained *FMR1* gene. For FSHD1 analysis, genomic DNA molecules aligning to regions of interest in chromosomes 4 and 10 were extracted and assembled. The resulting consensus maps were used to predict D4Z4 repeat size and assigned genotype of the permissive and non-permissive alleles (4qA and 4qB, respectively).

#### 2.2.2. Chromosomal Microarray Analysis

DNA was extracted from peripheral blood or amniotic fluid cells directly using QIAamp DNA Mini Kit (Qiagen, Hilden, Germany). CMA was conducted using Affymetrix CytoScan 750K array (Affymetrix, Santa Clara, CA, USA) following the manufacturer’s instruction, as previously reported [11]. Data analysis was performed using ChAS software (Ver. 4.2). Aberrations detected by CMA were classified into pathogenic, likely pathogenic, VOUS, likely benign and benign, following ACMG guidelines [10].

#### 2.2.3. Other Classic Cytogenetics and Molecular Genetics Technology

Karyotyping, FISH, MLPA and TP-PCR were all conducted following the standard procedures of the diagnostic laboratory, as previously described [12,13,14,15]. The results of G-banding karyotyping were described following the standards of the International System for Human Cytogenetic Nomenclature (ISCN) 2020 [16]. The fluorogenic-labeled probes of FISH were selected according to the results of CMA, karyotyping or OGM. After counterstaining chromosomes with fluorochromes, the super-resolution fluorescence microscope was used to observe fluorescent signals. MLPA was performed using SALSA MLPA Kits (MRC-Holland, Amsterdam, The Netherlands), and data were analyzed with Coffalyser.net software v.140721.1958 (MRC-Holland, Netherlands). TP-PCR was performed using AmplideX PCR/CE FMR1 PCR Kit (Asuragen, Austin, TX, USA) according to the manufacturer’s instruction.

## 3. Results

### 3.1. Cohort Description and Aberrations Missed by OGM

Our study included OGM data from 123 samples, of whom 109 were cultured amniotic fluid cell samples, and 14 were peripheral blood samples. According to the results of SOC methods, there were 154 clinically reported variations, including one triploidy, 31 aneuploidies, 95 CNVs and one ROH detected by CMA; five microscopic balanced translocations, one microscopic unbalanced translocation, two pericentric inversions and three isochromosomes detected by karyotyping; seven intragenic CNVs detected by MLPA; five submicroscopic balanced translocations and two submicroscopic unbalanced translocations detected by FISH; and one FMR1 gene full mutation detected by TP-PCR (Table S1). Taking GRCh37 and GRCh38 as reference genomes, the overall false-negative rates of OGM were 6.5% (10/154) and 4.5% (7/154), respectively. The 10 aberrations missed by OGM were summarized in Table 1.

### 3.2. Quality Control Performance

All the samples passed pre-analytical and analytical quality control. The quality control metrics of the 10 cases with aberrations missed by OGM well exceeded the set targets (Table 2): average total DNA of 525.277 Gb, average N50 (>150 kbp) of 279.206 kbp, average map rate of 91.47%, average label density of 15.42 labels per 100 kbp and average effective coverage of 149.719X. No significant difference was observed between the quality control metrics of the 10 cases with aberrations missed by OGM and those of the other 113 cases (*p* > 0.05).

### 3.3. Sample Type Analysis

A total of 16 clinically reported variations from 14 peripheral blood samples and 138 clinically reported variations from 109 amniotic fluid cell samples were identified by SOC methods. Taking GRCh37 as the reference genome, the false-negative rates of OGM in postnatal and prenatal settings were 18.8% (3/16) and 5.1% (7/138), respectively. There was no significant difference between the false-negative rates of OGM for peripheral blood samples and amniotic fluid cell samples (*p* = 0.117).

### 3.4. Reference Genome Analysis

Taking GRCh37 as the reference genome, OGM failed to identify 10 chromosomal aberrations, including five CNVs, two submicroscopic balanced translocations, two pericentric inversions of chromosome 9 and one isodicentric Y-chromosome mosaicism (7%). The overall false-negative rate of OGM was 6.5% (10/154). Taking GRCh38 as the reference genome, three CNVs (Case 2, 3 and 4) missed by OGM with the GRCh37 assembly were detected. The overall false-negative rate of OGM fell to 4.5% (7/154). Table 3 presented the false-negative rates of OGM for detecting different classes of variations, taking GRCh37 and GRCh38 as reference genomes, respectively.

### 3.5. Effective Coverage Analysis

To further analyze the influence of effective coverage on OGM results, we reran the de novo genome assembly of Case 1 and Cases 5–10 with the original BNX file, aiming at improving effective coverage. The results of Cases 5–10 with the higher effective coverage were consistent with previous results with the recommended effective coverage (80X). The result of Case 1 turned into ogm[GRCh38] 15q11.2(21,828,570–23,353,180)×1 with a confidence of 0.55.

### 3.6. Type and Location analysis

In our study, the variations missed by OGM involved CNVs, inversions, submicroscopic balanced translocations and isodicentric Y-chromosome mosaicism. All the other classes of variations were detected by OGM successfully.

#### 3.6.1. CNVs

**Figure 2:**
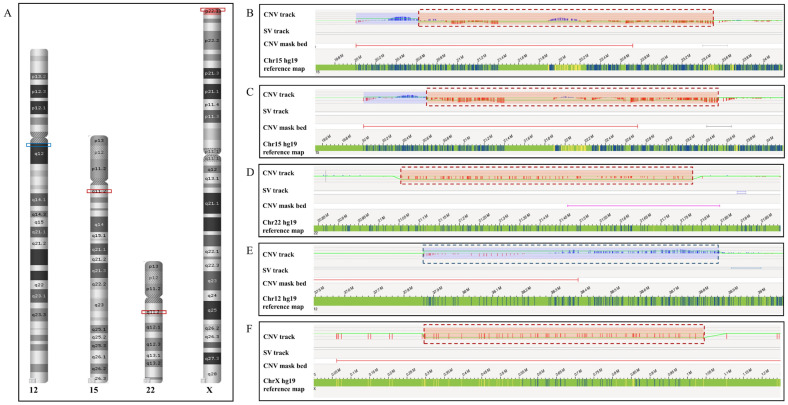
Summary of the five CNVs missed by OGM. (**A**) The location distribution of CNVs missed by OGM. The blue box represents duplication, and red boxes represent deletions. (**B**–**F**) OGM maps of Cases 1–5. (**B**–**F**) presented the two 15q11.2 deletions, one 22q11.2 deletion, one 12q11q12 duplication and one Xp22.33 deletion, respectively. OGM visualization map showed the five CNVs (dashed box) all involved CNV-masked region of OGM CNV algorithm. CNV: copy number variation; SV: structural variation.

Among the five CNVs missed by OGM, three (Case 1–3) were microdeletions located in recurrent CNV regions, including two 15q11.2 microdeletions and one 22q11.2 microdeletion; one was a terminal deletion of chromosome Xp22.33 with one breakpoint located in the subtelomeric region; the remaining one was interstitial microduplication of chromosome 12q11q12 with one breakpoint falling in the pericentromeric region (Figure 2). The five CNVs all involved CNV-masked regions of the OGM CNV algorithm, which meant that the breakpoints of these CNVs were located in SD regions or regions with no DLE-1 label (Figure 2B–F).

Additionally, not all the 15q11.2 deletions could be detected by OGM. There were, in total, eight 15q11.2 microdeletions, one 22q11.2 microdeletions, one Xp22.33 microdeletion and one 12q11q12 microduplication in our cohort, of which six 15q11.2 deletions were identified by the OGM CNV algorithm with high confidence (≥0.95). Taking GRCh37 and GRCh38 as reference genomes, the false-negative rate of OGM for 15q11.2 deletion detection was 25.0% and 12.5%, respectively. The OGM results of the eight cases of 15q11.2 deletion were presented in Figure S1.

#### 3.6.2. Balanced Translocations

OGM failed to detect two cryptic balanced translocations (Cases 6 and 7). The two cases both underwent abnormal pregnancy. Karyotyping of Cases 6 and 7 were both normal. Further testing by FISH was conducted to detect cryptic translocations according to the CMA results of the affected offspring or fetus.

**Figure 3:**
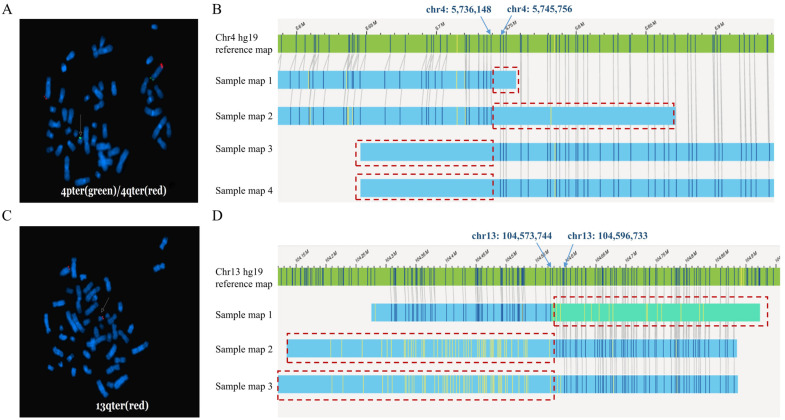
FISH results and OGM SV maps of Cases 6 and 7. (**A**) The FISH result of Case 6. FISH suggested ish t(4;?21)(4p−,4q+;21p+). (**B**) OGM SV maps of the translocation breakpoint of Case 6. (**C**) The FISH result of Case 7. FISH suggested t(13;21)(13q+,21q+). (**D**) OGM SV maps of the translocation breakpoint of Case 7. The red dashed boxes present the regions that cannot be mapped to another chromosome.

In Case 6, FISH showed that 4pter was translocated to another chromosome, which was speculated to be 21pter (Figure 3A). OGM failed to determine the translocation. Nevertheless, a breakpoint located at chr4: 5,736,148–5,745,756 (hg19) could be identified by manual inspection. The location of the breakpoint was concordant with the CMA result of the affected fetus, which suggested arr[hg19]4p16.3p16.2(68,345–5,746,086) × 3. However, OGM could not identify another breakpoint. As was shown in Figure 3B, there was hardly any label that could be mapped to another chromosome. Sample maps demonstrated that another breakpoint may have fallen in the region with incomplete reference sequences.

In Case 7, FISH suggested that 13qter was translocated to 21pter, confirming the presence of cryptic translocation (Figure 3C). OGM presented the breakpoint on chromosome 13 by manual review. The breakpoint detected by OGM was consistent with the CMA result of the affected offspring (13q33.1q34 deletion). But OGM was not able to identify the other breakpoint. (Figure 3D).

#### 3.6.3. Pericentric Inversion

There were two pericentric inversions of chromosome 9 that were undetected by OGM. One was inv(9)(p12q21) and the other was inv(9)(p12q13). The inverted regions covered the centromere, where no map was generated, and were flanked on either side by SD regions where SV maps might be misassembled.

#### 3.6.4. Isochromosome

Three cases of isodicentric Y-chromosome were included in our study. In Case 10, karyotyping quantified the mosaic ratio of 45,X and isodicentric Y-chromosome to be 93% and 7%, respectively. OGM can only identify 45,X; it cannot detect the low percent mosaic isodicentric Y-chromosome.

## 4. Discussion

CMA has been widely used to detect unbalanced chromosomal aberrations. It is recommended as the first-tier technology for the evaluation of individuals with congenital anomalies and fetuses with structural anomalies [10]. However, balanced translocations and inversions are out of the detection range of CMA. Although karyotyping is broadly implemented to detect balanced chromosomal rearrangements, it is not able to identify chromosomal anomalies smaller than 5 Mb due to the limitation of the resolution. FISH can be performed to detect cryptic chromosomal rearrangements according to the results of other genetic tests, such as CMA and karyotyping. Nevertheless, it is impossible for FISH to conduct breakpoint analysis. As is shown in Table S1, different SOC methods have advantages in identifying different classes of variations. Thus, sometimes multiple genetic methods should be performed simultaneously or in a step-wise fashion to detect SVs comprehensively.

OGM is a promising technology that can identify major classes of SVs in a single assay. It has been reported that OGM not only has advantage in detecting intragenic CNVs and cryptic and/or complex chromosomal rearrangements but can also perform breakpoint analysis, which is vital for interpretation of the clinical significance of SVs [17,18]. OGM has begun to be applied in prenatal and postnatal settings due to these significant advantages. It is well-known that SVs with centromeric breakpoints (such as Robertsonian translocations) are out of the detection range of OGM. However, there are also some other SVs that cannot be detected by OGM. It is important to study the SVs possibly being missed by OGM and analyze the contributing factors. Here, we conducted a study on a cohort of 123 individuals with 154 clinically reported variations to assess the detection range of OGM and explore the contributing factors. We described all the false-negative results and analyzed quality control metrics, sample types, reference genomes, effective coverage and types and distributions of these aberrations. Overall, OGM failed to detect 10 (6.5%, 10/154) chromosomal aberrations taking GRCh37 as the reference genome, including five CNVs, two submicroscopic balanced translocations, two pericentric inversion and one isochromosome (mosaicism). Compared with previous studies, the concordance rate in our study was slightly lower [5,6,19]. The main reason is that previous studies excluded the aberrations with (peri)centromeric breakpoints or VAF < 5%. When all the cases are included in a calculation, the false-negative rate of previous studies are similar to that of our study (Table S2). Additionally, because limited variations were available for the OGM test, a lot of CNVs were not included in previous studies. Therefore, more data and experience need to be accumulated. Our study summarized the false-negative results of OGM, emphasized the detection scope of OGM, and hoped to remind the public of the limitation of OGM.

Currently, users can analyze OGM data taking GRCh37 and GRCh38 as reference genome. Up to now, no study has analyzed the impacts of reference genomes on OGM results. No standard has been established regarding which reference genome is recommended to be used. In our study, we performed OGM, taking both GRCh37 and GRCh38 as reference genomes. According to our results, the performance of OGM with GRCh38 assembly is better than that of OGM with GRCh37 assembly, especially in the analysis of CNVs. The possible reason is that GRCh38 assembly provides more accurate and more complete genomic sequences and representations of population genomic diversity [20]. A previous study demonstrated that, compared with GRCh37, GRCh38 improved genome assembly and yielded more reliable genomic analysis results in the high-throughput sequencing data analysis [21]. Based on our results, the same is true in OGM data analysis. However, there were still some variations that were not able to be detected by OGM with GRCh38 assembly. In 2022, the Telomere-to-Telomere (T2T) Consortium addressed the remaining 8% gap of the genome, modified the reference genome and presented a complete assembly for all chromosomes except chromosome Y [22]. Currently, OGM is able to conduct de novo genome assembly taking T2T as the reference genome. Because the T2T reference genome perfected the complex regions of the genome, including centromere, telomere, SDs and short arms of acrocentric chromosomes, OGM data analysis will definitely benefit from T2T assembly. On one hand, a more complete genomic sequence means more DLE-1 labels throughout the genome; it will help CNV algorithm, which relies on quantifying digital molecules, to call CNVs. On the other hand, T2T assembly would enable the SV algorithm to assemble a more accurate and complete labelled genomic pattern, especially in (peri)centromeric, telomeric and SD regions. Although there is still no internal control database with T2T assembly in the Bionano Access system, we are looking forward to the performance of OGM taking T2T as a reference genome in the near future.

OGM was thought to be an all-in-one genetic test that can detect all the SVs with non-centromeric breakpoints. However, there were some classes of SVs (such as CNVs, pericentric inversions and submicroscopic balanced translocations) that were also missed by OGM in our study. According to the OGM maps, we speculated that these SVs had breakpoints in repetitive sequences or regions with no DLE-1 label. Actually, there were similar SVs undetected by OGM in previous studies. Dremsek P et al. [7] reported that OGM failed to detect a paracentric inversion of chromosome 8p23.1 and a deletion of chromosome Xp22.33. Researchers found that the inverted region of chromosome 8p23.1 was flanked on either side by SDs, and no OGM map spanned these SD regions. Therefore, the inversion was not confirmed. Additionally, OGM failed to identify the Xp22.33 deletion because of the poor coverage of this region. These reasons apply to our false-negative cases. In our study, there was also one case of Xp22.33 deletion that was not detected by OGM. All five false-negative CNVs in our study involved CNV-masked regions. In CNV-masked regions, poor genome assembly led to poor coverage. The repetitive sequences of these regions made it difficult for the SV algorithm to compare the sample’s labelled pattern to the reference genomic pattern accurately. The other CNVs reported undetected by OGM include 19p13.3 duplication, Xq28 deletion and 15q15.3 deletion [8]. As is shown in Table 1, the 22q11.2 deletion detected by the OGM SV algorithm in Case 3 was filtered due to the high frequency (16.2%) in the control database. It is important to further increase the sample size of the internal control database and establish a local database to improve the efficacy of OGM and help interpret SVs. In addition, based on the results of our study and previous studies, pericentric inversion of chromosome 9 is out of the detection range of OGM [6]. The principal reason is that the inverted region of chromosome 9 was flanked by large repetitive sequences and there was no OGM map generated in the centromere region. Furthermore, considering the high resolution of OGM, it was recommended as the first-line technology for cryptic chromosomal rearrangement [18,23,24]. However, it is worth noting that it is hard for OGM to call chromosomal rearrangement if the breakpoints are located in large repetitive sequences. In this situation, different technologies, including FISH, karyotyping and CMA, still need to be applied to complement OGM results.

To our knowledge, this is the first study focusing on the false-negative results of OGM and discussing the contributing factors of false-negative results. The limitation of our study is that the sample size was moderate. We will continue to increase the sample size in the future.

## 5. Conclusions

In conclusion, our results demonstrate that OGM may fail to identify SVs (such as CNVs, balanced translocations and inversions) with breakpoints located in large repetitive sequences besides Robertsonian translocations. GRCh38 is recommended as the reference genome when the OGM genome assembly is performed. When quality control metrics meet the set targets, increasing sequencing depth has limited influence on decreasing the false-negative rate. The detection range and limitation of OGM should be fully understood before OGM is used in clinical settings. Other genetic methods could be combined with OGM to improve the detection rate when necessary. T2T assembly may bring new opportunities for OGM in the near future.

## Figures and Tables

**Table 1:** Summary of the 10 aberrations missed by OGM.

Case	Sample Type	Results of SOC Methods	Clinical Significance	Results of OGM (GRCh37 as Reference Genome)	Results of OGM (GRCh38 as Reference Genome)
1	Cultured amniotic fluid cells	CMA: arr[hg19]15q11.2(22,770,421–23,191,761) × 1	VOUS	CNV algorithm: ogm[GRCh37]15q11.1q11.2(20,618,416–23,506,059) × 1 but filtered because confidence was 0	CNV algorithm: ogm[GRCh38]15q11.2(21,828,570–23,348,707) × 1 but filtered because confidence was 0.3
2	Cultured amniotic fluid cells	CMA: arr[hg19]15q11.2(22,770,421–23,281,886) × 1	VOUS	CNV algorithm: ogm[GRCh37]15q11.1q11.2(20,638,317–23,506,059) × 1 but filtered because confidence was 0	Detected CNV algorithm: ogm[GRCh38]15q11.2(22,076,863–23,396,245) × 1
3	Cultured amniotic fluid cells	CMA: arr[hg19]22q11.21(21,058,888–21,464,764) × 1	LP	CNV algorithm: ogm[GRCh37]22q11.21(21,054,185–21,779,032) × 1 but filtered because confidence was 0.93 SV algorithm: ogm[GRCh37]22q11.21(21,035,325–21,627,655) × 1 but filtered because frequency was 16.2% in control database	Detected CNV algorithm: ogm[GRCh38]22q11.21(20,699,897–21,424,743) × 1 SV algorithm: ogm[GRCh38]22q11.21(20,681,037–21,273,366) × 1
4	Cultured amniotic fluid cells	CMA: arr[hg19]12q11q12(38,012,530–39,027,084) × 3	VOUS	CNV algorithm: ogm[GRCh37]12q11q12(37,867,370–38,865,742) × 3 but filtered because FCN was 2.797	Detected CNV algorithm: ogm[GRCh38]12q11q12(37,261,973–38,471,358) × 3
5	Cultured amniotic fluid cells	CMA: arr[hg19]Xp22.33(168,551–881,102) × 1	P	CNV algorithm: ogm[GRCh37]Xp22.33(295,720–1,042,875) × 1 but filtered because confidence was 0	CNV algorithm: ogm[GRCh38]Xp22.33(544,608–1,439,001) × 1 but filtered because confidence was 0
6	Peripheral blood	FISH: ish t(4;?21)(4p−,4q+;21p+) Karyotyping: normal	-	Not detected	Not detected
7	Peripheral blood	FISH: ish t(13;21)(13q+,21q+) Karyotyping: normal	-	Not detected	Not detected
8	Cultured amniotic fluid cells	Karyotyping: 46,XN,inv(9)(p12q21)	-	Not detected	Not detected
9	Peripheral blood	Karyotyping: 46,XN,inv(9)(p12q13)	-	Not detected	Not detected
10	Cultured amniotic fluid cells	Karyotyping: 45,X [93]/46,X,idic(Y)(q11.22)[7]	P	CNV algorithm: 45,X Not detected idic(Y) mosaicism	CNV algorithm: 45,X Not detected idic(Y) mosaicism

SOC: standard-of-care; VOUS: variants of uncertain significance; P: pathogenic; LP: likely pathogenic; OGM: optical genome mapping; CNV: copy number variation; SV: structural variation; FCN: fractional copy number.

**Table 2:** Quality control metrics of the 10 cases with aberrations missed by OGM.

Case	Total DNA (≥150 kbp) (Gb)	N50 (≥150 kbp) (kbp)	Map Rate (%)	Average Label Density (N/100 kbp)	Effective Coverage (X)
1	517.81	259.88	87.8	15.30	142.27
2	528.85	267.38	92.9	15.09	152.27
3	526.96	278.65	92.0	15.64	152.02
4	513.97	233.31	88.9	15.13	141.39
5	545.90	288.75	94.0	15.34	158.95
6	556.44	289.73	94.2	15.55	164.64
7	522.08	265.88	93.0	15.58	150.76
8	527.47	312.46	95.2	15.41	155.35
9	504.78	285.09	92.9	15.40	145.16
10	598.51	312.80	85.2	15.76	135.28

**Table 3:** False-negative rates of OGM for detecting different classes of variations, taking GRCh37 and GRCh38 as reference genomes.

Clinically Reported Variations	Aberrations Detected by SOC Methods	Taking GRCh37 as Reference Genome		Taking GRCh38 as Reference Genome		*p* Value ^†^
		Aberrations Missed by OGM	False-Negative Rate of OGM	Aberrations Missed by OGM	False-Negative Rate of OGM	
Triploidy	1	0	0.0%	0	0.0%	-
Aneuploidy	31	0	0.0%	0	0.0%	-
Copy number variation	102	5	4.9%	2	2.0%	0.442 ^‡^
Balanced translocation	10	2	20.0%	2	20.0%	1.000 ^§^
Microscopic	5	0	0.0%	0	0.0%	-
Submicroscopic	5	2	40.0%	2	40/0%	1.000 ^§^
Unbalanced translocation	3	0	0.0%	0	0.0%	-
Microscopic	1	0	0.0%	0	0.0%	-
Submicroscopic	2	0	0.0%	0	0.0%	-
Pericentric inversion	2	2	100.0%	2	100.0%	1.000 ^§^
Isochromosome	3	1	33.3%	1	33.3%	1.000 ^§^
ROH	1	0	0.0%	0	0.0%	-
*FMR1* full mutation	1	0	0.0%	0	0.0%	-
Total	154	10	6.5%	7	4.5%	0.454 ^¶^

^†^ *p* value of false negative rates of OGM taking GRCh37 as reference genome versus false-negative rates of OGM taking GRCh38 as reference genome. ^‡^ Continuity correction. ^§^ Fisher’s exact test. ^¶^ Pearson chi-square. OGM: optical genome mapping; SOC: standard of care; ROH: regions of homozygosity.

## Data Availability

All the data generated or analyzed in this study are included in this published article.

## Supplementary Materials

The following supporting information can be downloaded at: https://www.mdpi.com/article/10.3390/diagnostics14020165/s1, Figure S1: Summary of the eight cases of 15q11.2 deletion in our study; Table S1: Results of standard-of-care methods and OGM for detection of different classes of variations; Table S2: Variations undetected by OGM in our study and in previous studies.
