# Supplementary material for: Optical Genome Mapping for Chromosomal Aberrations Detection—False-Negative Results and Contributing Factors
Source: Diagnostics (Basel). 2024 Jan 11;14(2):165. doi: 10.3390/diagnostics14020165 (PMC10814618; doi:10.3390/diagnostics14020165)
Supplement: Supplementary file 1 [file diagnostics-14-00165-s001.zip › diagnostics-2785576-supplementary.pdf]

Table S1. Results of standard-of-care methods and OGM for detection of different classes of variations †.

Clinically Reported Variations	Number	Standard-of-Care Methods, *n* (%)					OGM ‡, *n* (%)
		CMA	Karyotyping	MLPA	FISH	TP-PCR	
Triploidy	1	1 (100.0%)	1 (100.0%)	-	-	-	1 (100.0%)
Aneuploidy	31	31 (100.0%)	31 (100.0%)	-	-	-	31 (100.0%)
Copy number variation	102	-	-	-	-	-	100 (98.0%)
CNV > 50 kb	95	95 (100.0%)	9 (9.5%)	-	-	-	93 (97.9%)
CNV < 50 kb	7	-	-	7 (100.0%)	-	-	7 (100.0%)
Balanced translocation	10	-	-	-	-	-	8 (80.0%)
Microscopic	5	-	5 (100.0%)	-	-	-	5 (100.0%)
Submicroscopic	5	-	-	-	5 (100.0%)	-	3 (60%)
Unbalanced translocation	3	-	-	-	-	-	3 (100.0%)
Microscopic	1	-	1 (100.0%)	-	-	-	1 (100%)
Submicroscopic	2	-	-	-	2 (100.0%)	-	2 (100.0%)
Pericentric inversion	2	-	2 (100.0%)	-	-	-	0 (0.0%)
Isochromosome	3	-	3 (100.0%)	-	-	-	2 (66.7%)
ROH	1	1 (100.0%)	-	-	-	-	1 (100.0%)
FMR1 full mutation	1	-	-	-	-	1 (100.0%)	1 (100.0%)

† CMA, karyotyping and OGM were performed in all cases. MLPA was only applied in seven cases to detect intragenic CNVs. FISH was only performed in seven cases to identify submicroscopic translocations. TP-PCR was only used in one case to uncover *FMR1* full mutation. ‡ OGM was performed taking GRCh38 as reference genome.

Table S2. Variations undetected by OGM in our study and in previous studies.

Variations	Our Study †			Sahajpal N.S. et al. [6]			Mantere T. et al. [5]		
	n	Variations Missed by OGM	Rate	*n*	Variations Missed by OGM	Rate	*n*	Variations Missed by OGM	Rate
Triploidy	1	0	0.0%	3	0	0.0%	0	0	-
Aneuploidy	31	0	0.0%	27	1	3.7%	11	0	0.0%
Mosaic	0	0	-	5	1	20.0%	4	0	0.0%
Not mosaic	31	0	0.0%	22	0	0.0%	7	0	0.0%
Copy number variation	102	2	2.0%	57	0	0.0%	39	0	0.0%
Translocation	13	2	15.4%	6	5	83.3%	40	6	15.0%
Robertsonian	0	0	-	4	4	0.0%	2	2	100.0%
Other	13	2	15.4%	2	1	50.0%	28	4	14.3%
Inversion	2	2	100.0%	5	5	100.0%	6	0	0.0%
Paracentric	0	0	-	0	0	-	6	0	0.0%
Pericentric	2	2	100.0%	5	5	100.0%	0	0	0.0%
Isochromosome	3	1	33.3%	4	0	0.0%	6	0	0.0%
Mosaic	2	1	50.0%	4	0	0.0%	5	0	0.0%
Not mosaic	1	0	0.0%	0	0	-	1	0	0.0%
ROH	1	0	0.0%	6	0	0.0%	0	0	-
Insertion	0	0	-	0	0	-	2	0	0.0%
Complex rearrangement	0	0	-	0	0	-	4	2	50.0%
Ring chromosome	0	0	-	0	0	-	1	0	0.0%
FMR1 full mutation	1	0	0.0%	0	0	-	0	0	-

† OGM was performed taking GRCh38 as reference genome. OGM: optical genome mapping; ROH: regions of homozygosity.

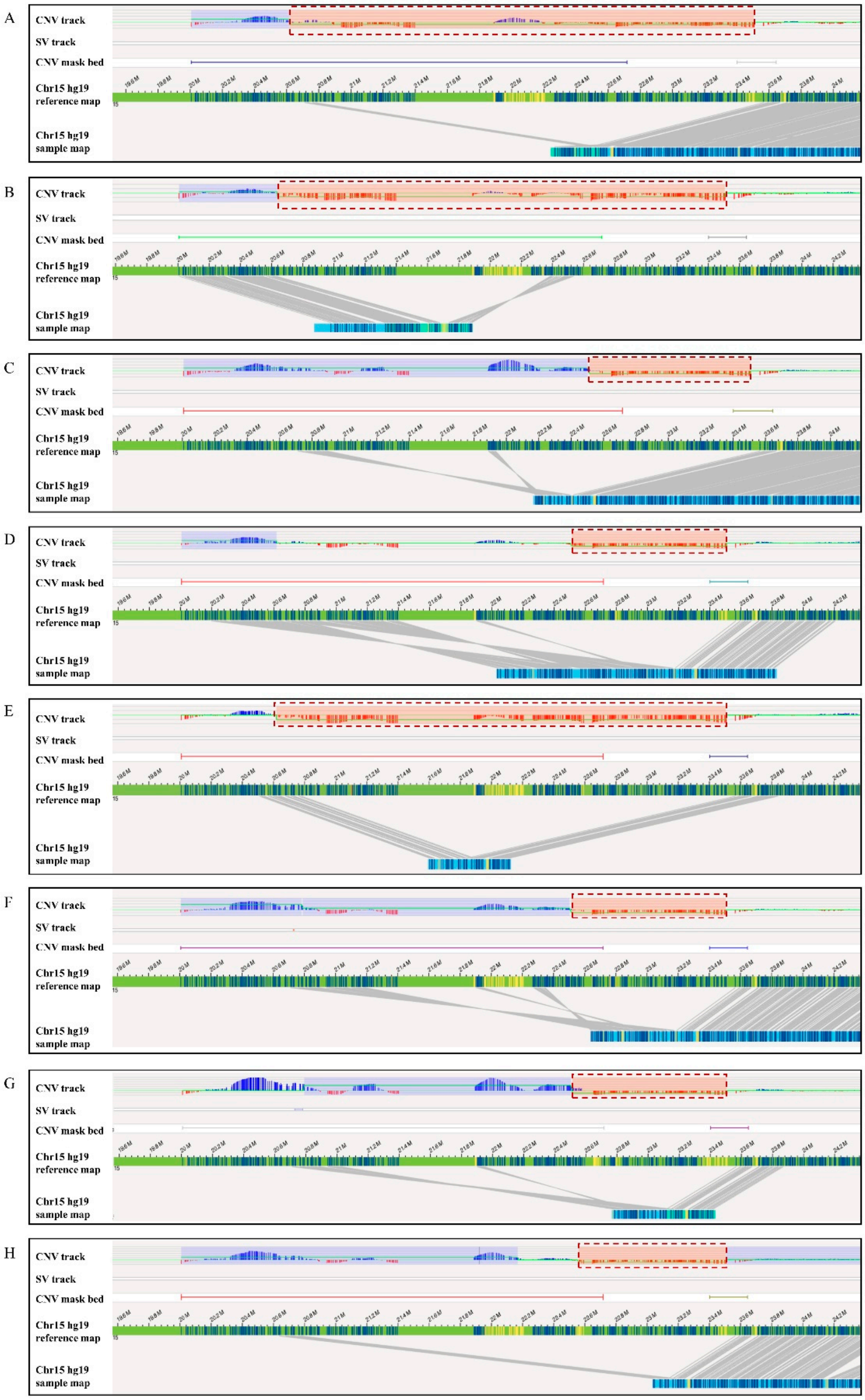

Figure S1. Summary of the eight cases of 15q11.2 deletion in our study. (**A**, **B**) OGM visualization maps of the two 15q11.2 deletions missed by OGM (Cases 1 and 2). (**C**–**H**) OGM visualization maps of the six 15q11.2 deletions detected by OGM. The red dashed boxes highlight the CNV locations call by OGM CNV algorithm. CNV: copy number variation; SV: structural variation.
